# Criteria of the German Society of Cardiology for the establishment of chest pain units: update 2014

**DOI:** 10.1007/s00392-015-0888-2

**Published:** 2015-07-07

**Authors:** Felix Post, Tommaso Gori, Evangelos Giannitsis, Harald Darius, Stephan Baldus, Christian Hamm, Rainer Hambrecht, Hans Martin Hofmeister, Hugo Katus, Stefan Perings, Jochen Senges, Thomas Münzel

**Affiliations:** Katholisches Klinikum Koblenz Montabaur, Koblenz, Germany; II. Medizinische Klinik und Poliklinik für Kardiologie, Angiologie und internistische Intensivmedizin, Universitätsmedizin Mainz, Langenbeckstr. 1, 55131 Mainz, Germany; Klinik für KardiologieAngiologie und Pneumonologie, Heidelberg, Germany; Department für Kardiologie, Innere Medizin und Intensivmedizin, Vivantes-Klinikum Neukölln, Berlin, Germany; Klinikum III für Innere Medizin Uniklinik Köln, Cologne, Germany; Kerkhoff-Klinik, Bad Nauheim, Germany; Klinik für Kardiologie und Angiologie, Herzzentrum Bremen, Bremen, Germany; Kardiologie und allgemeine Innere Medizin, Solingen, Germany; Cardiozentrum, Düsseldorf, Germany; Institut für Herzinfarktforschung, Ludwigshafen, Germany

**Keywords:** Chest pain, Certification, Requirements network guidelines

## Abstract

Since 2008, the German Cardiac Society (DGK) has been establishing a network of certified chest pain units (CPUs). The goal of CPUs was and is to carry out differential diagnostics of acute or newly occurring chest pain of undetermined origin in a rapid and goal-oriented manner and to take immediate therapeutic measures. The basis for the previous certification process was criteria that have been established and published by the task force on CPUs. These criteria regulate the spatial and technical requirements and determine diagnostic and therapeutic strategies in patients with chest pain. Furthermore, the requirements for the organization of CPUs and the training requirements for the staff of a CPU are defined. The certification process is carried out by the DGK; currently, 225 CPUs are certified and 139 CPUs have been recertified after running for a period of 3 years. The certification criteria have now been revised and updated according to new guidelines.

## Introduction

**Fig. 1:**
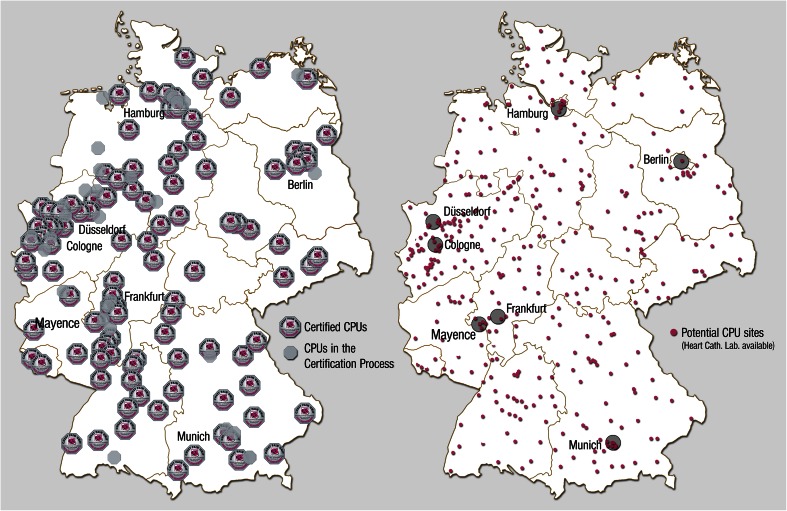
Certified CPUs, CPUs in certification process and potential CPU sites in Germany 2014

In 2008, the German Society of Cardiology (Deutsche Gesellschaft für Kardiologie–Herz- und Kreislaufforschung, DGK) [1] defined the criteria for the establishment of chest pain units (CPUs). The scope of this manuscript was to define minimum criteria for a CPU that was to be valid nationwide. Institutions that already ran a CPU were also given the possibility, through a continuous evaluation and re-evaluation process, to take advantage of technical innovations. Accordingly, a certification program was initiated in 2008; to date, 200 CPUs have been certified based on the criteria of the DGK, and 134 of these have already renewed their certification (Fig. 1) [2].

Using the same criteria, CPUs were also certified in Zurich and Lucerne in Switzerland. Furthermore, a consensus paper of the DGK defining the criteria to expand this process to private clinics was published in 2010 [3]; a certification process has since been established for the private sector and 30 private institutions have been certified to date. CPUs have received attention in national and international guidelines [4, 5].

The general goal of a CPU was and is to carry out in a rapid and goal-oriented manner differential diagnosis of acute or newly occurring chest pain of undetermined origin. Data from similar processes in the USA and UK [6–9] demonstrate the superiority of CPUs compared with standard emergency care units. These data also show that the establishment of CPUs leads to a reduction in hospitalization times and a reduction in costs [10–12] due to the better utilization of diagnostic and therapeutic methods [8, 10–12]. Finally, the establishment of CPUs also improves patient satisfaction [13].

Through their participation in a national registry [14], certified CPUs also participate in a network whose scope is to collect clinically and academically relevant data on the epidemiology, treatment, and outcome of patients with chest pain. The first data from this registry have already been published [15, 16]. The criteria for the certification of CPUs have been revised by the DGK “Chest Pain Unit Task Force” to replace the original publication from 2008 [1]. In the current, revised version, changes in the diagnosis of acute chest pain during the past 2 years have served as a basis for subsequent new certifications as well as re-certifications.

The basic requirements, such as the availability of a cardiac catheterization laboratory around the clock, remain basically the same as originally stipulated in 2008. The experiences collected in these 6 years, and during the re-certification process, as well as recent scientific findings and new guidelines, however, require that this position paper be revised.

## Space requirements

In terms of infrastructure, a CPU must be allocated at least four beds, all equipped with heart rhythm and blood pressure monitoring capabilities. These beds have to be under the clinical and organizational management of a cardiologist. They can be located in a separate spatial unit or be integrated into a central internal medicine facility or emergency room; however, the area of the CPU must be precisely identified and designated. The capacity must be sufficient for monitoring multiple patients over a period of at least 6–8 h. The exact number of beds can vary based on the size of the expected patient volume, taking into account sufficient reserves for situations with high patient volumes. As a minimum standard, however, four beds are to be present to qualify a unit as a CPU. Since the experience of recent years shows that the patient load can be high, it seems reasonable to plan at least one additional bed per 50,000 inhabitants in the region being served. A system that guarantees that sufficient flexible reserves can be allocated to the CPU for emergency or overflow situations must also be in place. Additional rooms for patient consultations, diagnostic instrumentation, ambulant patients, and patients’ relatives are desirable.

The CPU must be integrated in the emergency system of the hospital (including in-house resuscitation and emergency teams) (see Table 1).

**Table 1:** Spatial requirements for the establishment of a CPU

Criterium	Minimum requirement	Additional DGK recommendation
Rooms	Integration in an emergency unit with continuous availability of defined facilities (see below), led by cardiologists	Well-designated rooms, monitoring room, waiting room, treatment room, conference room
Bed capacity	At least four monitored beds	1 additional bed per 50,000 inhabitants in the region
Access	24 h a day/7 days a week^a^	
Catheterization laboratory	In-house, continual access (24/7)^a^	
Resuscitation/emergency concept	The CPU must be integrated in the in-house emergency concept (emergency team)	

^a^Except in cases where there are technical issues

## Technical requirements

A CPU must meet the basic technological requirements for the diagnosis of acute or recent onset chest pain of unclear origin. It has to be allocated a 12-lead ECG [4] and systems for rhythm monitoring, non-invasive blood pressure measurement, and pulse oximetry at each bedside [17, 18].

Transthoracic echocardiography by a trained examiner must be available on site within 30 min, 24 h a day, 7 days a week (24/7), for the diagnosis of wall motion abnormalities, heart defects, right heart failure, and pericardial effusion. Transesophageal echocardiography should also be available on site [19, 20].

Standard emergency care infrastructure must be available. This includes both a fully equipped emergency unit (with a defibrillator, airway intubation equipment, oxygen, and a suction device) as well as the capacity to transport unstable patients (including ECG monitor, infusion pump, transportable ventilator). The emergency equipment must be checked regularly and be in line with the current state of the art.

Twenty-four-hour access to emergency laboratory diagnostics is required. The time from blood collection to delivery of the results must not exceed 45–60 min; it should be checked regularly that this interval remains within these limits [4]. If this is not the case, a Point-of-Care Test Unit (POCT) for the measurement of cardiac biomarkers should be available in the CPU [4]. Results of ischemic markers must be quantitative (as compared with positive/negative). Blood gas analysis should be available within 15 min.

Availability of instruments and trained personnel for the analysis of internal cardioverter/defibrillators (ICD) and pacemakers should be guaranteed 24/7 with a response time of less than 6 h. Percutaneous pacemaker therapy should be available.

A multi-slice CT must be on hand for further investigation of relevant differential diagnoses after exclusion of acute coronary syndrome (pulmonary embolism, aortic dissection) or to rule out coronary artery disease of low or intermediate probability following pretest. Based on risk stratification, patients with suspected coronary artery disease without unstable characteristics (e.g., those who are free of symptoms, without primary or secondary risk indicators) may be discharged, but a system that guarantees re-admission for further investigation within three business days (or any time earlier in case of symptom relapse) must be in place. This system may also be implemented in cooperation with external private or public walk-in clinics (see Table 2).

**Table 2:** Technical requirements

Criterium	Minimum requirement	Additional recommendation by the DGK
12-lead ECG	Permanent availability	
Blood pressure measurement	At each bed	Non-invasive blood pressure monitoring in the waiting room, facilities for implementing invasive monitoring
TTE	Available 24/7, response time <30 min	Dedicated CPU machine
Rhythm monitoring	At each bed	
Resuscitation	Dedicated facilities, including defibrillator	
Transportation with ECG monitoring	Permanently available (if necessary with equipment from the intensive care unit)	CPU-dedicated devices
Transport ventilator	Permanently available (if necessary with equipment from the intensive care unit)	CPU-dedicated devices
Laboratory diagnostics	24-h availability; turn-around time 45–60 min	POCT, turn-around time <20 min
Blood gas analysis	Available; turn-around time <15 min	Integration in the CPU
External pacemaker	Permanently available (if necessary with equipment from the intensive care unit)	CPU-dedicated devices
Exercise stress test, CT	Available within three business days; an appointment must be given upon discharge	Cooperation with external walk-in clinics

*TTE* transthoracic echocardiography, *POCT* Point-of-Care Testing, *CT* computed tomography

## Diagnostic procedures

National and international guidelines for the diagnosis of acute chest pain must be implemented and observed [4, 19, 21, 22].

A 15-lead ECG (including standard and posterior leads V7 to V9) must be recorded immediately upon admission of each patient [4], and this ECG must be evaluated by a physician within 10 min [4]. It is reasonable to record right precordial leads in each patient with inferior myocardial infarction, as this may have prognostic and therapeutic implications. An ECG must be recorded again after 6 h or upon symptom recurrence [23, 24]. An additional ECG 3 h after admission is recommended in order bridge the 6-h gap between recordings, and this is also useful for patients who can be discharged early in an accelerated “rule-out protocol” using high-sensitivity troponin measurements.

In addition to the clinical assessment and ECG, the diagnosis of acute coronary syndrome always includes the assessment of cardiac markers. Cardiac troponins, ideally high-sensitivity troponin T or I, should be preferred as they have the highest sensitivity and can show an irreversible myocardial necrosis [23, 24]. It is recommended that troponin levels be checked at admission and 6–9 h thereafter [4] (this interval can be reduced to 3 h if high-sensitivity troponin is used) [23, 24]. An increasing number of studies show that strategies such as the use of a threshold for troponin below the 99th percentile [25, 26], the shortening of the intervals between tests to 60–120 min [27, 28], or the use of other biomarkers such as copeptin in combination with troponin allow an earlier diagnosis of acute coronary syndrome [29] and a safe early discharge in case these biomarkers are negative [30]. CPUs exposed to a high volume of patients might particularly profit from such strategies. In addition, an early diagnosis of non-ST-segment elevation myocardial infarction (NSTEMI) has clinical implications for patients and allocation of resources [31]. The determination of other biomarkers may be useful depending on the clinical diagnosis. Determination of D-dimer levels can be used to rule out acute pulmonary embolism or acute aortic syndrome in patients with unexplained chest pain [19, 21].

Non-cardiac baseline parameters must be recorded upon admission, including a full blood count, electrolytes, creatinine, CRP, glucose, and coagulation status. Thyroid function parameters (particularly basal TSH) are optional but may be important in case there is a need for subsequent contrast media exposure or in patients with known or suspected thyroid disease. Arterial blood gas analysis should be carried out only if there is explicit clinical indication.

A transthoracic echocardiography is performed as clinically indicated; this includes all patients with suspected acute coronary syndrome or suspected aortic dissection [in the latter case transesophageal echocardiography (TEE), computed tomography, (CT), or magnetic resonance imaging (MRI) should be employed] [4, 31]. An ultrasound machine equipped with an appropriate probe and staff trained in performing an ultrasound of the abdomen should be available at all times.

In line with the indications of the ESC and the DGK, scoring systems, e.g. the GRACE score [32], should be used to improve and standardize the risk stratification of the patients [33]. Accordingly, high-risk patients (GRACE score >140 points) should undergo coronary angiography within 24 h; those patients who are at moderate or lower risk should undergo angiography within 72 h [34]. The GRACE score is determined using eight independent risk parameters that include age, heart rate, and ST-segment abnormalities. If the GRACE score is below 108 points, the risk of patients dying in the hospital is less than 1 %. A moderate score of 109–140 points is associated with medium risk (1–3 %). Patients with 141–372 points show an in-hospital mortality rate of more than 3 % [34]. The use of alternative or additional scoring systems is advisable [35–37] (see Table 3).

**Table 3:** Diagnostic strategies in the CPU

Criterium	Minimum requirements	Additional DGK recommendation
Cardiac biomarkers	Troponin T or I	hsTroponin T, BNP, Nt-proBNP, Copeptin
Time points of biomarker assessments	0 and 6–9 h after admission	0–3 h When hsTroponin T is assessed and at symptom recurrence; 0–1 (2) h hsTn assays in patients at low risk
Blood sampling (general)	Electrolytes, creatinine, full blood count, CRP, coagulation, D-Dimer if clinically indicated	Additional biomarker panel, including thyroid function test
Time point of blood sampling	At admission	Based on clinical indication
ECG	12-lead ECG recorded and interpreted within 10 min. Additional leads (V_3_r, V_4_r, V_7_ to V_9_) can be useful to detect ischaemia that frequently escapes the common 12-lead ECG	V_3_r, V_4_r, V_7_ to V_9_ at all time points
Time point of ECG	0 + 6 h after admission and at symptom recurrence	0–3–6 After admission and at symptom recurrence
TTE	All patients with suspected ACS, available 24/7	
Risk stratification	GRACE score at admission	Additional risk scores
Exercise test	All patients after exclusion of ACS	In cooperation with external partners
Abdominal ultrasound	Available 24/7 in cooperation (e.g. with emergency services)	In the CPU

*CK* creatine kinase, *BNP* B-type natriuretic peptide, *hs-Troponin T* high-sensitivity troponin T, *TTE* transthoracic echocardiography, *ACS* acute coronary syndrome

## Therapy

A CPU is designed to optimize the diagnostic processes and therapeutic options in patients with chest pain. Each CPU must establish and implement strict standard operating procedures (SOPs) for the following diseases:

- ST-elevation myocardial infarction (STEMI)
  Use of different SOPs based on patient presentation (e.g., hemodynamic stability/instability, referral from emergency services or self-referral)
- NSTEMI
- unstable angina pectoris
- stable angina pectoris
- hypertensive crisis
- acute pulmonary embolism
- acute aortic diseases
- cardiogenic shock
- decompensated heart failure
- resuscitation
- ICD discharge
- pacemaker dysfunction
- atrial fibrillation

These treatment recommendations do not necessarily dictate that ACS patients should undergo triage to be treated exclusively in the CPU. Especially in cases of STEMI and cardiogenic shock, patients should be transferred directly from the ambulance to the catheterization laboratory [22]. These SOPs must nonetheless be well structured and defined.

Transfer times from CPU to catheterization laboratory in the case of high-risk patients should never exceed 15 min.

At the time of discharge, patients must receive a discharge letter including recommendations for therapy, especially in case of symptom relapse [4, 21, 22]. In addition, every patient should participate in a documented and structured consultation concerning lifestyle modifications (smoking cessation, exercise, and diet) and risk factors of medical therapy (LDL-cholesterol target values) (see Table 4).

**Table 4:** Therapeutic strategies in the CPU

Criterium	Minimum requirement	Additional recommendation
Algorithms	STEMI (different SOP for self-referral and referral through emergency service), NSTEMI, unstable angina pectoris, stable angina pectoris, hypertensive crisis, acute pulmonary embolism, acute aortic syndrome, atrial fibrillation, cardiogenic shock, resuscitation, ICD discharge, pacemaker dysfunction, atrial fibrillation	Additional algorithms
Catheterization laboratory	Each STEMI: within 90–120 min (contact-to-balloon time) or according to current guidelines Each NSTEMI/UA: <24 h after admission for high-risk patients (GRACE > 140), within 72 h for intermediate risk patients, or according to guidelines	
STEMI program	Direct transfer to catheterization laboratory	

*STEMI* ST-elevation myocardial infarction, *NSTEMI* Non-STEMI, *UA* unstable angina pectoris, *SAP* stable angina pectoris

## Diagnostic algorithms for patients with suspected acute coronary syndrome and low risk

An early risk stratification is of paramount importance to triage patients into groups requiring immediate (<120 min), early (<24 h), or delayed (<72 h) invasive diagnostics or to allocate them to more conservative therapy. Patients without primary or secondary risk characteristics that remain free of symptoms during the course of admission and examination can be discharged early. A previous meta-analysis of eight studies showed that use of early invasive diagnostics leads to a 22 % reduction in the composite endpoint of death, myocardial infarction, or hospitalization for ACS [4]. Patients who are positive for biomarkers (cTn, hsTn), i.e. NSTEMI patients, profit particularly from this invasive approach [38], while patients with negative biomarkers do not profit from it, and women with negative biomarkers actually show a worse prognosis when exposed to unnecessary invasive exams [39]. The discharge of a patient after an accelerated diagnostic process based on the assessment of both cardiac troponin and copeptin appears to be as safe as the standard protocol with a repeated troponin assessment after 6 h [30]. In patients at low risk (GRACE score <108 or TIMI 0–1) such accelerated diagnostic algorithms allow the ruling out of NSTEMI with two troponin assessments in the normal range within 60–120 min. As long as both values remain below the 99th percentile, the negative predictive value of such an approach is greater than 99 % [28].

The ESC Guidelines also recommend against performing routine cardiac catheterization in asymptomatic patients without risk characteristics, especially changes in high-sensitivity troponin T values or an ischemic ECG (level of evidence IIIC). Therefore, the decision to direct a patient to invasive investigations should be based on the results of laboratory tests, ECG, and exercise (stress) tests. Stress tests should be carried out either before discharge or shortly thereafter (≤3 working days).

In patients with low or intermediate pre-test probability for the presence of acute coronary syndrome, multi-slice CT angiography is recommended to rule out coronary artery disease ([4], level of evidence IC).

Primary risk criteria

- Relevant rise or drop of cardiac troponin
- Dynamic ST- or T-wave changes
- GRACE score >140

Secondary risk criteria

- Diabetes mellitus
- Kidney failure (eGFR <60 ml/min/1.73 m^2^)
- Reduced LV ejection fraction (<40 %)
- Early post-infarct angina
- History of PCI or ACVB
- Intermediate to high GRACE risk score (http://www.gracescore.org)

## Cooperations

A cardiac catheterization laboratory with permanent personnel available for acute intervention is an indispensable prerequisite for a CPU. The catheterization laboratory must be on duty 24/7; the only allowed exception is unexpected technical failure, in which case the facility may be temporarily logged out of the emergency care program. The reasons for such lapses must be recorded and a fail-safe concept must be present. Permanent staff availability must be guaranteed and should be documented by means of service plans; here also a fail-safe concept is required.

Of central importance is a close cooperation with the regional emergency care facilities and emergency structures, and these should not be negatively affected by the establishment of a CPU. For patients with STEMI who are diagnosed prior to arrival at the hospital, a fast-track protocol should be defined that bypasses the CPU and leads directly to the catheterization laboratory. Referring and emergency physicians should be offered the opportunity of a telemedical ECG transmission online or via fax [40].

An important in-hospital interface must exist with an intensive care unit or an intermediate care ward. The transfer time must not exceed 15 min.

Facilities must be in place to allow conventional X-ray diagnoses and CT scans, and it should be possible to consult with specialists in other disciplines in-house or in cooperation with external partners.

In addition, a strong link to external walk-in clinics must be established. This cooperation should also be extended to prevention and awareness campaigns. If an outpatient chest pain clinic exists, a collaboration should be sought (see Table 5).

**Table 5:** Cooperations und partners of a CPU

Criterium	Minimum requirement	Additional recommendation
General emergency room	Available 24/7	In the same building (but separate room facilities)
Emergency outpatient clinic	Integration of the CPU in the existing emergency structures	Development of an integrated regional and transregional model
Emergency physician	Preclinical STEMI program with direct transfer of the patient to the catheterization laboratory	
Intensive care unit	Available 24/7; transfer time <15 min	Integration of CPU, ER, and ICU in a complex model
Catheterization laboratory	Available 24/7, transfer <15 min	
Radiology	Chest X-ray (available 24/7) CT (available 24/7)	Cardio-MRI, scintigraphy within 3 days
Additional cooperations	Cardiovascular and thoracic surgery	Other medical specialties

*MRI* magnetic resonance imaging

## Education

The nursing staff must undergo special training. A specific training program for “Chest Pain Unit Nurse”, certified by the DGK, has been established. Standard emergency training is also obligatory and should be repeated at least twice per year [41].

Members of the medical staff should be able to demonstrate 2 years of professional experience in internal medicine, echocardiographic knowledge, and sufficient experience in internal intensive care medicine. CPU doctors are not necessarily allocated exclusively to this unit, but their shift must be organized in a way so as to guarantee the presence of a physician within 10 min of patients´ admission and in case of need (e.g. parallel work in the emergency service ward is not allowed). A consultant specialized in cardiology must be on call with a maximum response time of 30 min. Each patient must be seen by a specialist before discharge. These requirements must be met at any time of the day or night, including holidays.

Each employee must be thoroughly informed about the standard operating procedures and trained in dealing with patients with acute chest pain. The local operating procedures must be based on international guidelines and must be documented in writing. All employees must undergo regular resuscitation training (Advanced Life Support). It may be useful to integrate local emergency services in the training programs to improve the entire chain of lifesaving procedures for acute or new-onset chest pain.

A report must be made at regular intervals (preferably quarterly), the results of which should be documented in team meetings and case conferences. Feedback mechanisms should also be introduced that reflect the results and the quality of treatment and diagnosis. Every patient should be informed in a structured manner about the necessary lifestyle changes (quitting smoking, performing regular exercise, engaging in healthy eating) and the importance of a medical therapy in preventing future cardiovascular events (see Table 6).

**Table 6:** Education and training of the CPU

Criterium	Minimum requirements	Additional recommendation
Physicians	At least 2 years internal medicine/cardiology experience, adequate intensive care experience, echocardiography training	
Consultant	Cardiologist	Continuous presence of a specialist in the CPU
Nurses	Special CPU training	“CPU Nurse” title
Training	Emergency training at least twice a year, case conferences	
Quality control	Feedback mechanisms for the quality of the diagnosis and therapy	Participation in the CPU registry

## Organization

A CPU is part of a cardiology department or clinic that provides for the possibility to administer invasive coronary therapy. If the beds of a CPU are associated with an emergency department, they must be expressly designated as CPU beds that are part of a cardiological facility. A cardiologist must be responsible for the management of the CPU, and his/her response time shall not exceed 30 min.

One physician (or physician-in-training) must be constantly present in the CPU. The ratio between patients and nurses should not exceed 4:1, so that at least two nurses must be present if the number of monitored patients exceeds four.

Since a CPU is an emergency unit, it cannot be closed at any time (see Table 7).

**Table 7:** Organization of a CPU

Criterium	Minimum requirement	Additional recommendation
Supervision	Specialist in cardiology	
Physician	Continual presence	Shift system guaranteeing the continual presence of a qualified staff member
Consultants (cardiologists)	On call 24/7; response time <30 min	Continual presence
Nurses	Present 24/7; maximally a 4:1 patient-to-nurse ratio	

## The certification process

Application for certification may be made at the office of the DGK. An invoice for the certification fee will be sent to the applying institution; payment of the first half of the amount is due 14 days after the invoice is sent and is a prerequisite for further action by the DGK. The application process begins formally with the mailing of the invoice.

After payment, the applicant receives an electronic data entry form saved on a CD-Rom. This is to be completed by the applicant and returned.

The DGK then informs the committee for the certification of CPU, which suggests the names of two independent, trained referees for the assessment of the application; if they are approved they are invited by the committee to review the application.

The expert referees next contact the applicant and arrange an appointment for an audit. After the audit, the experts write a report and a recommendation, which are sent to the DGK. The committee decides on the basis of these documents whether or not to grant the CPU certification.

Based on the evaluation, the DGK issues either a certification (“CPU–DGK certified” logo), a rejection (with justification), or a certification pending fulfillment of conditions [42].

A certification is valid for 3 years, after which the CPU needs to be re-certified for another 5 years. The re-certification process is similar to the initial certification process but only involves one expert referee.

## Perspective

An overview of the current changes in the criteria of the DGK for the certification and re-certification process of CPUs is provided in Table 8.

**Table 8:** Relevant changes of the criteria of the German Society of Cardiology for Chest Pain Unit: 2008 to 2014

Criterium	2008 Minimal requirements	Additional recommendations	2014 Minimal requirements	Additional recommendations
Rhythm monitoring	At each bed	ST-segment monitoring	At each bed	*ST-segment monitoring omitted*
Exercise testing, CT-scan coronary arteries	Available within three business days; an appointment must be given upon discharge and entered in the discharge letter; when possible, in cooperation with outpatient clinics	Located in the CPU	Available within three business days; an appointment must be given upon discharge and entered in the discharge letter	*When possible, in cooperation with outpatient clinics*
Laboratory values (cardiac)	Troponin T or I	CK, CK-MB, BNP, nt-proBNP, multimarker, Myoglobin	Troponin T or I	hsTroponin T, BNP, Nt-proBNP, *Copeptin (new) (multimarker and myoglobin omitted)*
Timing of determination of laboratory values	0 + 6 to 12 h after admission	0–3–6 h, additional sampling after another chest pain event	0 + 6 *and up to 9* *h* after admission	*0–3* *h in case of use of hsTroponin T, additional sampling after another Chest Pain Attack, 0–1 (2)* *h in case of hsTn assays in patients with low risk*
Laboratory values (general)	Electrolytes, creatinine, blood count, CRP, coagulation status	Additional diagnostics, thyroid function tests (TSH), (repeated) D-Dimer if clinically indicated	Electrolytes, creatinine, blood count, CRP, coagulation status, *D-Dimer if clinically indicated* (*now minimal requirement*)	Additional diagnostics when indicated, thyroid function tests (TSH)
TTE	All unstable patients, based on clinical indications. Available on 365 days/24 h		*All patients with ACS or other clinical indications—365* *days/24* *h availability*	
*Risk stratification* (*new*)			*GRACE-score at admission*	*Additional risk scores*
Algorithms for patients` treatment	STEMI (two different algorithms for patients with in-hospital and pre-hospital diagnosis), NSTEMI, unstable angina pectoris, stable angina pectoris, hypertensive crisis, acute lung embolism, acute aortic dissection, cardiogenic shock, resuscitation	Additional algorithms	STEMI (two different algorithms for patients with in-hospital and pre-hospital diagnosis), NSTEMI, unstable angina pectoris, stable angina pectoris, hypertensive crisis, acute lung embolism, acute aortic dissection, cardiogenic shock, resuscitation, *ICD discharge, SM-malfunction, atrial fibrillation (new)*	Additional algorithms
Catheterization laboratory accessibility	Every STEMI within 90–120 min, every NSTEMI/UA with moderate to high risk 48–72 h		Every STEMI within 90–120 min (*contact-to-balloon time*), patients with NSTEMI/UA *with very high risk: immediately; with high risk* (*GRACE* *>* *140*) *within 24* *h; with low risk: within 72* *h,* or according to most current guidelines	
Emergency services	Integration in the regional plan for ACS		*Integration in the existing emergency system*	*Integrated structures for the therapy of ACS at a regional and nationwide*
Catheterization laboratory	Available 365 days/24 h, transfer time <15 min, with at least four interventional cardiologists		Available 365 days/24 h, transfer time <15 min (*the criterion of at least four cardiologists is deleted*)	
Additional cooperations	Gastroenterology, heart surgery, outpatient clinics	Psychosomatic medicine	Heart surgery, outpatient clinics, *gastroenterology is omitted*	Other disciplines *Psychosomatic medicine is omitted*
Nursing staff	Presence: 365 days/24 h	Intensive care unit training	*Special CPU training*	*Accredited training as Nurse expert (Chest Pain Unit),* intensive care unit training
*Quality control*			*Feedback mechanisms for the assessment of the quality of the diagnostic and therapy*	*Participation in the CPU-registry*

Changes are highlighted in italic

To date, 200 CPUs have been certified in Germany and more than 134 CPUs re-certified. This rapid growth underscores the interest in the advantages that this structure offers. The number of CPUs in Germany already far exceeds that of the rest of Europe. The objective of our initiative remains to achieve nationwide coverage through a network of certified CPUs throughout the country. To meet this goal, it will be necessary to certify as many as 300 CPUs, as to date there are significant regional differences in cardiological care. Furthermore, we aim to export the concept to a European level, a process that has already begun. The criteria for certification will need to be updated constantly following technical developments and innovations, and they must be based on the most current guidelines. The German CPU registry will also have a central importance in evaluating standards of care and treatment strategies [14], while single-center experiences already demonstrate the benefit associated with the establishment of a CPU. To date, 30,087 patients have been enrolled in the CPU registry since December 2008, and the first data have already been published [15, 16, 43–45].
